# Association of Lipoprotein and Apolipoprotein Ratios With Glycemic Levels in Individuals With Prediabetes: A Case-Control Study

**DOI:** 10.7759/cureus.63500

**Published:** 2024-06-30

**Authors:** Sherwan Salih

**Affiliations:** 1 Department of Medical Chemistry, College of Medicine, University of Duhok, Duhok, IRQ

**Keywords:** prediabetes, hba1c, lipoprotein, insulin resistance, apolipoprotein

## Abstract

Background: Prediabetes is defined as a hyperglycemic state between normal glucose metabolism and diabetes mellitus. It is also recognized as a predisposing factor for cardiovascular disease. Apolipoprotein is a constituent of lipoproteins, and its ratio levels (ApoB/ApoA1 ratio) are considered an independent risk factor for cardiovascular diseases. This study aimed to evaluate the apolipoprotein ratio (ApoB/ApoA1 ratio) and lipoprotein ratio (low-density lipoprotein cholesterol/high-density lipoprotein cholesterol (LDL-C/HDL-C) ratio) in prediabetes in relation to glycemic levels and establish the association between apolipoprotein and lipoprotein ratios in prediabetic individuals and their glycemic levels.

Methodology: A case-control study was conducted among 150 participants, 75 with prediabetes and 75 apparently healthy individuals (with no prediabetes or diabetes), from January 1, 2023 to December 30, 2023. The parameters involved are fasting serum glucose, insulin, blood HbA1c%, HDL-C, LDL-C, apolipoprotein A, apolipoprotein B, and lipoprotein(a) (Lp(a)), measured using different principles.

Results: Prediabetes was more predominant in males (58.7%), particularly those aged over 40 years (74.7%). The mean Lp(a) (46.18±11.66 mg/dl), LDL-C/HDL-C ratio (1.74±0.96), and ApoB/ApoA ratio (1.10±0.62) were significantly higher among prediabetic individuals. Moreover, these ratios were insignificantly higher in prediabetic individuals with HbA1c level (5.8-6.4%) and fasting glucose level (100-125 mg/dl) than those with lower levels.

Conclusions: Prediabetic individuals exhibited a notably elevated average level of Lp(a), as well as increased mean ApoB/ApoA1 ratio and mean LDL-C/HDL-C ratio compared to individuals who were apparently healthy.

## Introduction

Prediabetes is defined as a hyperglycemic state between normal glucose metabolism and diabetes mellitus and is classified as impaired glucose tolerance (IGT) and impaired fasting glucose (IFG) [1]. Although the majority of prediabetic individuals are asymptomatic, it can be considered as a predictor for the development of type 2 diabetes mellitus (T2DM) and cardiovascular diseases (CVDs) [2].

The low-density lipoprotein cholesterol/high-density lipoprotein cholesterol ratio (LDL-C/HDL-C ratio) has been recognized as a more precise indicator of cardiovascular events. Moreover, it has been demonstrated to function as a specific marker for lipid-lowering interventions linked to coronary heart disease [3].

Lipoprotein(a) (Lp(a)) is a variant of LDL and considered as an independent risk factor for CVD, as a study demonstrated that cardiovascular disorder risk increases proportionally with increasing Lp(a) levels [4]. Moreover, Lp(a) is highly associated with impaired glucose metabolism [5].

Apolipoproteins, integral constituents of lipoproteins, are affixed to the surface of lipoproteins and play a pivotal role in determining the properties, transport, and metabolism of lipoproteins [6]. Apolipoprotein B (ApoB), a vital element of very-low-density lipoproteins (VLDLs) as well as their derivatives, intermediate-density lipoproteins (IDLs) and LDLs, along with chylomicrons and their remnants, is encoded by the *APOB* gene. It is instrumental in maintaining the structural stability of lipoproteins [7].

Apolipoprotein A-I (ApoA-I), constituting the primary protein component of HDL (comprising approximately 70% of the protein content of HDL-C), is regulated by the human *ApoA1* gene. ApoA-I is well recognized for its role in regulating cholesterol trafficking and providing protection against CVD, initiation of cancer, and modulating inflammatory and immune responses [8]. ApoA-I is predominantly detected in the liver, small intestine, and colon [9].

However, to our current understanding, there have been limited studies that have identified an independent association between the ApoB/ApoA1 ratio and lipoprotein ratio with glycemic levels in individuals with prediabetes [10]. Consequently, the objective of this study is to evaluate the ApoB/ApoA1 ratio and LDL-C/HDL-C ratio in prediabetes in relation to glycemic levels and establish the association between apolipoprotein and lipoprotein ratios in prediabetic individuals and their glycemic levels.

## Materials and methods

A case-control study was conducted involving a total of 150 participants, comprising 75 individuals diagnosed with prediabetes and an equivalent number of apparently healthy individuals forming the control group. The study spanned a period of one year, commencing on January 1, 2023, and concluding on December 30, 2023. Individuals with prediabetes were identified from among the relatives of patients with DM attending the Diabetic and Endocrinology Unit at Azadi Teaching Hospital, Duhok City, Kurdistan region, Iraq. Control participants were selected from the pool of medical staff and college employees who were considered to be in good health, non-alcoholic, non-smokers, with fasting serum glucose (FSG) levels below 100 mg/dl and HbA1c levels below 5.8%. Exclusion criteria encompassed individuals with liver diseases, renal diseases, thyroid diseases, those on hypolipidemic or steroid medications, pregnant women, or individuals with any other conditions that could potentially impact the lipid profile under study.

Ethical approval for the study was obtained from the Duhok General Health Directorate and the College of Medicine of Duhok University under the reference number (27032024-2-11).

An interview session was organized to administer a questionnaire specifically designed to fulfill the study's criteria. The questionnaire encompassed inquiries pertaining to participants' names, ages (including date of birth), genders, and family histories of DM. Both the prediabetic and control groups were carefully matched in terms of age and gender (Appendix).

Anthropometric assessments were conducted, incorporating measurements of waist circumferences (measuring tape), weights (automated balance), and heights (measuring tape), all of which were utilized in the computation of the body mass index (BMI). The BMI was calculated by dividing the weight in kilograms by the square of the height in meters (kg/m²) [11].

The diagnosis of prediabetes depends on the American Diabetes Association as IFG having FSG levels 100-125 mg/dl or IGT having glucose level two hours post oral glucose tolerance test of 140-199 mg/dl with FPG less than that for the diagnosis of DM [6]. All the participants (prediabetic and control individuals) were informed to fast overnight and to attend the Clinical Biochemistry Department of Azadi Teaching Hospital in the morning for blood collection. Ten milliliters of blood were obtained from all participants and collected in two tubes (Gel tube and EDTA tube) for the measurement of biochemical parameters such as glucose concentration, insulin level, whole blood HbA1c%, ApoA, ApoB, and Lp(a), HDL-C, and LDL-C.

Glucose, insulin, HbA1c%, HDL-C, and LDL-C were measured using Cobas 6000 (Roche, Hitachi) based on different principles. Serum glucose concentration, HDL-C, and LDL-C were measured using the enzymatic-colorimetric method, insulin was measured by electrochemiluminescence, and blood HbA1c was was measured using the turbidimetric assay [12-14]. The serum levels of ApoA, ApoB, and Lp(a) were determined using the enzyme-linked immunosorbent assay (ELISA) method (Assay Genie kit, Europe). The devices involved were Cobas 6000 and ELISA, which were controlled daily at the beginning of the work by standard controls provided by the company that supplied the devices as well as a calibration curve. Homeostatic model assessment of insulin resistance (HOMA-IR) was calculated from glucose and insulin values using the formula: HOMA-IR = (fasting glucose in mg/dl × fasting insulin in µIU/mL)/405. Insulin resistance was considered present when HOMA-IR exceeded 2.5 [15].

Statistical analysis

The anthropometric attributes of both prediabetic and healthy control individuals were presented as mean values with standard deviations (SD) or as percentages. Independent t-tests and Pearson chi-square tests were employed to assess the comparative differences between the two study groups. The Pearson chi-square test was specifically utilized to evaluate predominant differences between the groups. A p-value of 0.05 or less was considered statistically significant. Statistical analyses were conducted using IBM SPSS Statistics for Windows, Version 26.0 (Released 2019; IBM Corp., Armonk, New York, United States).

## Results

General characteristics between prediabetic and healthy control individuals are shown in Table 1. The mean age of individuals with prediabetes was 43.11±5.48 years, with a male predominance (58.7%), and nearly three-quarters of them (74.7%) were above 40 years of age. Moreover, the mean WC (106.79±14.18 cm), BMI (33.9±5.7 kg/m^2^), and positive family history of diabetes (60.0%) were significantly higher among individuals with prediabetes compared to controls.

**Table 1:** General characteristics of study participants. BMI: body mass index; DM: diabetes mellitus. ^a^Pearson chi-square and ^b^independent t-tests were performed for statistical analysis. *Statistically significant.

Subject characteristics	Pre-DM, n=75	Controls, n=75	p-value
	Mean±SD, N (%)	Mean±SD, N (%)	
Gender, N (%)			
Male	44 (58.7%)	42 (56.0%)	0.741^a^
Female	31 (41.3%)	33 (44.0%)	
Age (years)	43.11±5.48	42.77±5.96	0.722^b^
<40	19 (25.3%)	21 (28.0%)	0.854
≥40	56 (74.7%)	54 (72.0%)	
Waist circumferences	106.79±14.18	92.88±33.89	<0.001^b*^
Male <102 cm	20 (23.3%)	34 (39.5%)	0.001^a*^
Male ≥102 cm	24 (27.9%)	8 (9.3%)	
Female <88 cm	2 (3.1%)	18 (28.1%)	<0.001^a*^
Female ≥88 cm	29 (45.3%)	15 (23.4%)	
BMI, kg/m^2^	33.9±5.7	24.6±3.4	<0.001^b*^
Normal, N (%)	2 (2.6%)	48 (64.0%)	<0.001^a*^
Overweight and obese, N (%)	73 (97.4%)	27 (36.0%)	
Family history of diabetes, N (%)			
Positive	45 (60.0%)	7 (9.3%)	<0.001^a*^
Negative	30 (40.0%)	68 (90.7%)	

Biochemical parameters of the studied group are shown in Table 2. The mean levels of glucose (104.92±4.27), HbA1c (5.77±0.39), insulin (16.63±10.38), and HOMA-IR (4.30±2.26) were significantly higher in individuals with prediabetes compared to healthy individuals. Moreover, Lp(a), LDL-C/HDL-C ratio, and ApoB/ApoA ratio were significantly higher among individuals with prediabetes.

**Table 2:** Biochemical parameters of study participants. HOMA-IR: hemostasis model assessment of insulin resistance; LDL/HDL-C: high-density lipoprotein cholesterol/low-density lipoprotein cholesterol ratio; ApoB/ApoA: apolipoprotein A/apolipoprotein B ratio; DM: diabetes mellitus. Independent t-test was performed for statistical analysis. *Statistically significant.

Characteristics	Pre-DM	Controls	p value
	n=75	n=75	
	Mean±SD	Mean±SD	
Glucose, mg/dl	104.92±4.27	82.29±9.67	<0.001^*^
Insulin, µIU/ml	16.63±10.38	12.27±3.77	0.001^*^
HOMA-IR	4.30±2.26	2.49±0.81	<0.001^*^
HbA1c%	5.77±0.39	4.93±0.45	<0.001^*^
HDL-C, mg/dl	52.27±13.54	59.36±19.16	0.010^*^
LDL-C, mg/dl	77.24±23.15	56.43±28.93	<0.001^*^
Lipoprotein(a), mg/dl	46.18±11.66	27.05±10.95	<0.001^*^
ApoA, mg/dl	105.77±45.68	115.09±36.95	0.347^*^
ApoB, mg/dl	126.43±64.76	118.36±36.22	0.171
LDL-C/HDL-C	1.74±0.96	1.29±0.79	0.002^*^
ApoB/ApoA	1.10±0.62	0.77±0.34	<0.001^*^

The mean levels of LP(a), lipoprotein ratio, and apolipoprotein ratio were insignificantly higher in prediabetic individuals with HbA1c between 5.8% and 6.4% compared to those with HbA1c <5.8% as shown in Table 3: Lp(a) (46.84±12.03 mg/dl), LDL-C/HDL-C ratio (1.99±1.05 mg/dl), and ApoB/ApoA ratio (1.11±0.64 mg/dl). 

**Table 3:** Lipoprotein and apolipoprotein ratio in relation to HbA1c% in individuals with prediabetes. LDL/HDL-C: high-density lipoprotein cholesterol/low-density lipoprotein cholesterol ratio; ApoB/ApoA: apolipoprotein A/apolipoprotein B ratio; DM: diabetes mellitus. Independent t-test was performed for statistical analysis. *Statistically significant.

Characteristics	Pre-DM HbA1C <5.8%	Pre-DM HbA1C 5.8-6.4%	p value
	N=9	N=66	
	Mean±SD	Mean±SD	
HDL-C, mg/dl	59.88±19.43	55.56±17.52	0.529
LDL-C, mg/dl	76.17±23.11	85.07±23.22	0.282
Lp(a), mg/dl	41.38±7.19	46.84±12.03	0.189^*^
ApoA, mg/dl	131.44±62.55	125.75±65.46	0.806
ApoB, mg/dl	107.98±35.91	116.07±37.25	0.541
LDL/HDL-C	1.70±0.94	1.99±1.05	0.394^*^
ApoB/ApoA	0.97±0.49	1.11±0.64	0.515^* ^

The relationship of Lp(a), lipoprotein ratio, and apolipoprotein ratio with glucose level in individuals with prediabetes is shown in Table 4. The mean levels of the serum Lp(a) (48.12±9.4), LDL-C/HDL-C ratio (2.11±0.97), and ApoB/ApoA ratio (1.12±0.65) were insignificantly higher in individuals with prediabetes with FSG between 100 and 125 mg/dl compared to those with FSG <100 mg/dl.

**Table 4:** Lipoprotein and apolipoprotein ratio in relation to glucose level in individuals with prediabetes. FSG: fasting serum glucose; LDL/HDL-C: high-density lipoprotein cholesterol/low-density lipoprotein cholesterol ratio; ApoB/ApoA: apolipoprotein A/apolipoprotein B ratio; DM: diabetes mellitus. Independent t-test was performed for statistical analysis. *Statistically significant.

Characteristics	Pre-DM FSG <100 mg/dl (IGT)	Pre-DM FSG 100–125 mg/dl	p value
	N=8	N=67	
	Mean ±SD	Mean ±SD	
HDL-C, mg/dl	60.28±18.8	51.63±21.51	0.229
LDL-C, mg/dl	76.67±23.64	81.97±19.08	0.544
Lp(a), mg/dl	45.95±11.93	48.12±9.4	0.622
ApoA, mg/dl	126.99±67.53	121.76±36.05	0.831
ApoB, mg/dl	110.86±18.94	115.61±38.59	0.731
LDL/HDL-C	1.69±0.95	2.11±0.97	0.240
ApoB/ApoA	0.96±0.22	1.12±0.65	0.503

The correlation of glycemic levels with lipoprotein, apolipoprotein ratio, and anthropometrics characteristics in individuals with prediabetes is shown in Table 5. There was a positive correlation of blood glucose with waist circumferences (r=0.236 and p=0.041). The levels of ApoB/ApoA ratio (r=0.256 and p=0.027) and waist circumferences (r=0.238 and p=0.039) were positively and significantly correlated with the HbA1c%. There was a positive and significant correlation of HOMA-IR with LDL/HDL-C ratio (r=0.241 and p=0.038).

**Table 5:** General and biochemical characteristics with glycemic levels in individuals with prediabetes. FSG: fasting serum glucose; HOMA-IR: hemostasis model assessment of insulin resistance; LDL/HDL-C: high-density lipoprotein cholesterol/low-density lipoprotein cholesterol ratio; ApoB/ApoA: apolipoprotein A/apolipoprotein B ratio; BMI: body mass index; DM: diabetes mellitus. Bivariate correlation was performed for statistical analysis. *Statistically significant.

Characteristics	FSG in pre-DM		HbA1c in pre-DM			HOMA-IR in pre-DM	
	n=75		n=75			n=75	
	Pearson correlation (r)	Sig. (two-tailed) (p value)	Pearson correlation (r)	Sig. (two-tailed) (p value)	Pearson correlation (r)		Sig. (two-tailed) (p value)
HDL-C, mg/dl	-0.083	0.480	-0.070	0.553	-0.228		0.050^*^
LDL-C, mg/dl	0.204	0.079	0.056	0.636	0.137		0.243
Lp(a), mg/dl	0.007	0.949	0.029	0.805	0.029		0.803
ApoA, mg/dl	-0.035	0.764	-0.161	0.167	-0.129		0.269
ApoB, mg/dl	-0.266	0.021^*^	0.256	0.027^*^	0.057		0.624
LDL/HDL-C	0.018	0.876	0.130	0.267	0.241		0.038^*^
ApoB/ApoA	-0.110	0.347	0.342	0.003^*^	0.149		0.201
Waist circumferences	0.236	0.041^*^	0.238	0.039^*^	0.060		0.608
BMI, kg/m^2^	0.107	0.362	0.208	0.073	0.128		0.275
Family history of diabetes	0.118	0.313	-0.044	0.708	-0.177		0.129

The relationship of HOMA-IR with lipoprotein and apolipoprotein ratio is shown in Table 6. The mean level of LDL-C/HDL-C ratio (2.34±0.98) was significantly higher, whereas ApoB/ApoA ratio was insignificantly higher in prediabetes individuals with HOMA-IR ≥2.5 compared to those with HOMA-IR <2.5.

**Table 6:** Lipoprotein and apolipoprotein ratio in relation to HOMA-IR in individuals with prediabetes. HOMA-IR: hemostasis model assessment of insulin resistance; LDL/HDL-C: high-density lipoprotein cholesterol/low-density lipoprotein cholesterol ratio; ApoB/ApoA: apolipoprotein A/apolipoprotein B ratio; DM: diabetes mellitus. Independent t-test was performed for statistical analysis. *Statistically significant.

Characteristics	Pre-DM HOMA-IR <2.5	Pre-DM HOMA-IR ≥2.5	p value
	N=10	N=65	
	Mean ±SD	Mean ±SD	
HDL-C, mg/dl	60.77±19.32	50.0±15.99	0.105
LDL-C, mg/dl	74.82±22.43	92.30±22.61	0.020^*^
Lp(a) mg/dl	44.48±9.05	46.45±12.05	0.616
ApoA, mg/dl	157.68±90.48	121.62±59.34	0.102
ApoB, mg/dl	108.61±28.89	157.23±61.69	<0.001^*^
LDL/HDL-C	1.64±0.92	2.34±0.98	0.031^*^
ApoB/ApoA	1.067±0.57	1.29±0.89	0.281

## Discussion

Prediabetes is one of the most prevalent but denied and unaware conditions in almost all over the world, as nearly 90% of adults currently living with prediabetes deny or are unaware of it [16]. Additionally, prediabetes is considered as a risk factor for the development of CVD [17]; therefore, it is important to manage and control their lipid profile, lipoprotein ratio levels, and apolipoprotein ratio levels.

Our data reinforce the concept that obesity and insulin resistance underline the pathogenesis of prediabetes. In this study, the observed results showed that prediabetes is more common in obese males over 40 years old with a positive family history of diabetes mellitus. Prediabetes is commonly associated with an abnormal increase in body weight; therefore, worldwide, the prevalence of prediabetes is increasing as the prevalence of obesity rises [18,19]. This is mostly related to insulin resistance and inflammation. These metabolic derangements cause endothelial vasodilator and fibrinolytic dysfunction, resulting in an increased risk of prediabetes complications, particularly cardiovascular disorders [20].

In this study, there was a significant association between prediabetes and mean lipoprotein, mean lipoprotein ratio, and mean apolipoprotein ratio in our population, independent of traditional metabolic risk factors. There was a significant high mean level of Lp(a), mean ApoB/ApoA1 ratio, and mean LDL-C/HDL-C ratio in individuals with prediabetes compared to apparently healthy individuals. ApoB levels were significantly increased in individuals with prediabetes, while the levels of ApoA-I showed the opposite trend. Consequently, the mean ratio of ApoB/ApoA-I was positively associated with prediabetes. This can be due to the increased production rates and decreased catabolism of intestinal ApoB48 and hepatic ApoB100 [18]. The present results are highly consistent with a study conducted in China that showed a strong association between the mean ApoB/ApoA1 ratio and prediabetes [21]. Furthermore, increased blood glucose concentration in prediabetes individuals interferes with normal lipid metabolism, causing glycation of apolipoproteins, leading to the appearance of dyslipidemia components [22].

Numerous research studies have highlighted the association between oxidative stress and the pathogenesis of insulin resistance, involving the inhibition of insulin signals and dysregulation of adipocytokines [18,23]. Several research suggested that Lp(a), lipoprotein ratio (LDL-C/HDL-C ratio), and apolipoprotein ratio (ApoB/ApoA-I ratio) have a higher association with CVD than with individual lipids [24-26]. Apolipoprotein A and apolipoprotein B play pivotal roles in regulating and sustaining the production and metabolism of lipoprotein particles. Consequently, the ApoB/ApoA ratio serves as an indicator of the equilibrium between atherogenic and atheroprotective particles. A higher ApoB/ApoA ratio is indicative of an increased propensity for cholesterol accumulation, thereby elevating the risk of CVD [27].

Dyslipidemia and abnormal glucose metabolism cluster as risk factors causing one another. HbA1c is considered as an important indicator of blood glucose control. This study observed that the Lp (a), ApoB/ApoA-I ratio, and LDL-C/HDL-C ratio were closely correlated with serum glucose, blood HbA1C, and HOMA-IR in individuals with prediabetes. Prediabetes with insulin resistance (decreased insulin function) results in decreased lipolysis process and reduced lipoprotein lipase activity. Therefore, after meal consumption, hepatic free fatty acid production increases, whereas HDL-C becomes smaller, has less antiatherogenic activity, and is easily removed from the circulation by the kidneys [28]. Consistent with these findings, our results align with a study conducted in a Chinese population, where obese individuals without diabetes exhibited the ApoB/ApoA ratio as an independent predictor of insulin resistance [28]. Additionally, Duez et al. demonstrated an elevated intestinal secretion of ApoB48-containing triglyceride in individuals with insulin resistance and hyperinsulinemia. A potential explanation for the observed positive correlation between the ApoB/ApoA-I ratio and insulin resistance may be their shared association with an inflammatory state [29]. The study has its own limitation: the study population included only individuals with prediabetes, who do not adequately represent the general population with impaired glucose metabolism.

A strong correlation was found between the ApoB/ApoA1 ratio and glycemic levels in individuals with prediabetes. This ratio could be used as a biomarker for the early detection and monitoring of prediabetes. This would facilitate timely interventions to prevent the progression to diabetes and reduce cardiovascular risks. Understanding the metabolic profiles associated with prediabetes can inform public health strategies aimed at reducing the burden of diabetes.

## Conclusions

The study concluded that prediabetes is more common in obese males over 40 years old with a positive family history of diabetes mellitus. Individuals with prediabetes exhibited significantly higher mean levels of Lp(a), mean ApoB/ApoA1 ratio, and mean LDL-C/HDL-C ratio compared to apparently healthy individuals. There was a close correlation between mean Lp(a), ApoB/ApoA-I ratio, and LDL-C/HDL-C ratio with serum glucose, blood HbA1C, and HOMA-IR in individuals with prediabetes. The study's findings could pave the way for longitudinal studies to track changes in lipoprotein ratios over time and their impact on diabetes progression and cardiovascular outcomes. Further longitudinal studies are needed to consider additional confounding factors, such as diet, physical activity, and genetic predisposition, and to determine the risky prediction of these lipoprotein and apolipoprotein ratios for diabetes and CVD.

## Figures and Tables

**Figure 1:**
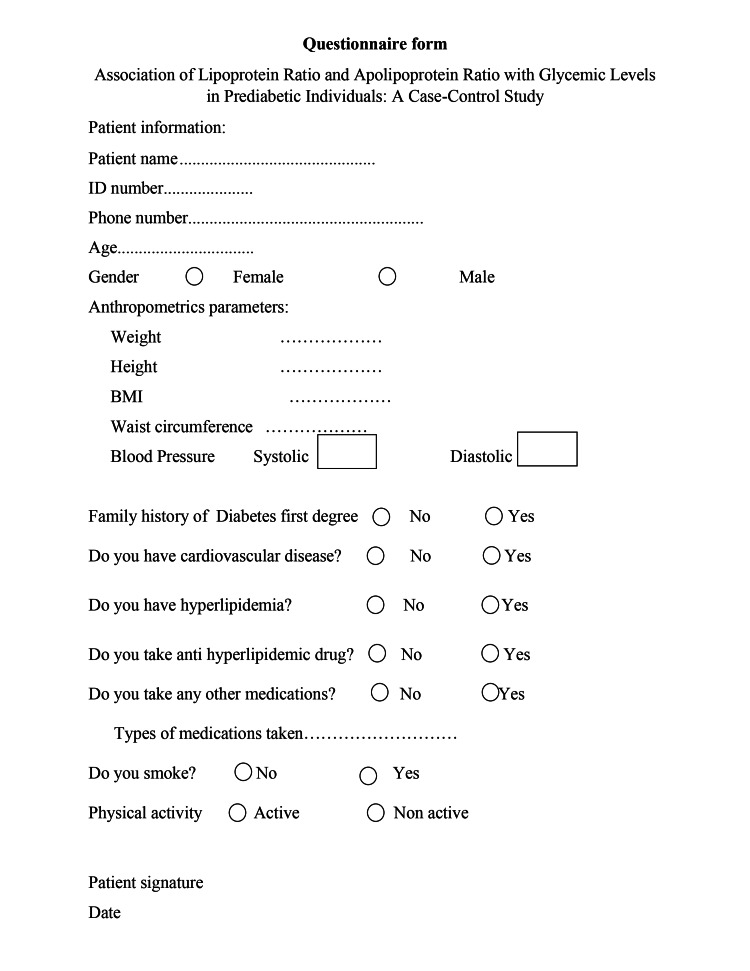
Questionnaire form. BMI: body mass index.
